# Does Olfactory Dysfunction Correlate with Disease Progression in Parkinson’s Disease? A Systematic Review of the Current Literature

**DOI:** 10.3390/brainsci12050513

**Published:** 2022-04-19

**Authors:** Tommaso Ercoli, Carla Masala, Gianluca Cadeddu, Marcello Mario Mascia, Gianni Orofino, Angelo Fabio Gigante, Paolo Solla, Giovanni Defazio, Lorenzo Rocchi

**Affiliations:** 1 Department of Medical Sciences and Public Health, Institute of Neurology, University of Cagliari, SS 554 km 4.500, 09042 Cagliari, Italy; g.luca.cadeddu@gmail.com (G.C.); giovanni.defazio@unica.it (G.D.); lorenzo.rocchi@unica.it (L.R.); 2 Department of Biomedical Sciences, University of Cagliari, SP 8 Cittadella Universitaria, 09042 Monserrato, Italy; cmasala@unica.it; 3 Institute of Neurology, Azienda Ospedaliero Universitaria di Cagliari, SS 554 km 4.500, 09042 Cagliari, Italy; marcello.mas@tiscali.it (M.M.M.); gianni.orofino@tin.it (G.O.); 4 San Paolo Hospital, Via Capo Scardicchio, 70123 Bari, Italy; angelo.gigante@yahoo.it; 5 Unit of Neurology, Department of Medical, Surgical and Experimental Sciences, University of Sassari, 07100 Sassari, Italy; psolla@uniss.it

**Keywords:** Parkinson’s disease, olfactory dysfunction, disease progression, smell, olfaction

## Abstract

Background. Loss of olfaction is a well-established early feature of Parkinson’s disease (PD). Although olfactory dysfunction has been widely described as a prodromal feature of PD in the literature, whether it can be considered a biomarker of PD progression is still a matter of debate. Objective. The aim of this work is to define the possible relationship between the progression of olfactory dysfunction and other putative clinical hallmarks of PD over time, through a systematic review of the current literature. Methods. We conducted a systematic review of the literature on PubMed from inception to March 2022. We included only longitudinal studies conducted on patients with diagnosis of idiopathic PD who underwent olfactory function testing at baseline and repeated it at least once during follow-up. Results. Among 5740 records identified through database searching, nine longitudinal studies met full criteria and underwent data extraction. Conclusions. Olfaction seemed to decrease over time, albeit with a degree of fluctuation. Moreover, smell detection ability seems to deteriorate more rapidly in the early phase of disease, indicating a possible association with disease progression. More studies are needed to better understand the role of olfaction as a biomarker of PD progression over time.

## 1. Introduction

Decreased olfactory function is a common phenomenon during aging, with hyposmia being present in over three quarters of subjects over the age of 80 years [1]. Several factors are probably involved in age-related loss of olfaction, including local issues (i.e., nasal congestion, cumulative damage to olfactory epithelium, and increased susceptibility to nasal diseases) and deterioration of the olfactory system (i.e., loss of selectivity of olfactory cells to odorants and abnormalities in central olfactory pathways, including structural and functional damage, as well as impairment in neurotransmission and modulatory systems involved in the synaptic regulation of olfactory circuitry) [1,2,3].

Besides being common in healthy, elderly individuals, loss of olfaction is a well-established early feature of Parkinson’s disease (PD), neurodegenerative diseases such as Lewy Body Dementia, Alzheimer’s disease, Multiple System Atrophy, and other movement disorders [4,5,6,7,8,9,10,11,12,13]. Although its role as a prodromal feature of PD has been widely investigated in the literature, the progression of olfactory dysfunction after a diagnosis of PD has not been thoroughly assessed. Many cross-sectional studies have found a correlation between smell impairment and disease duration in PD; however, retrospective cross-sectional studies may be open to recall, cause-and-effect, and misclassification bias. Therefore, it is not clear whether olfactory loss may be considered as a marker of PD progression. If this hypothesis were true, olfactory function should be expected to decrease at a significantly faster rate than in normal aging and to be correlated with other markers of PD progression. Of note, olfactory dysfunction has been reported to be independent from anti-parkinsonian therapy [14,15].

The mechanism underlying olfactory dysfunction in PD is still not well defined, and both peripheral and central olfactory pathways seem to be involved. The anterior olfactory nucleus and the olfactory bulb have been considered the first sites of PD onset in previous studies [16,17], and the presence of α-synuclein aggregates has been described in the olfactory bulb at early stages of the disease. However, α-synuclein aggregates have also been reported in the piriform cortex, the amygdala, the olfactory tubercle, the entorhinal cortex, and the orbitofrontal cortex [18,19,20]. This staging model correlates the spread of α-synuclein aggregates in the central nervous system over disease progression with PD symptoms. Indeed, early stages correspond to non-motor symptoms (such as olfactory dysfunction), mid-stages to motor impairment, and later stages to cognitive decline [16].

The identification of reliable markers of progression is an unmet need in PD [21] and current putative biomarkers might not sufficiently mirror PD progression, due to the possible confounding role of dopaminergic medication [22]. So far, clinical trials have often relied on subjective and/or rater-dependent measures, which might be less suited to assess the efficacy of novel and/or disease-modifying therapies for PD, compared to objective/semi-objective ones. This systematic review thus aimed at defining whether olfactory detection ability can capture longitudinal PD progression by examining longitudinal studies that addressed this issue.

## 2. Methods

We performed a systematic review of the literature on PubMed from inception to March 2022 using the following searching string: (((((((((olfactory *) OR smell) OR hyposmia) OR microsomia) OR anosmia) OR odor) OR phantosmia) OR sense) OR olfaction) AND ((Parkinson’s disease) OR Parkinson disease). The reference list of each selected article was checked to screen for additional studies possibly worth including, but not captured by the original search method. We conducted a systematic review following the Preferred Reporting Items for Systematic Reviews and Meta-analyses (PRISMA) guidelines [23].

We included only longitudinal studies carried out in patients with a diagnosis of idiopathic PD who underwent any type of olfactory testing at baseline and repeated it at least once during follow-up. Only studies referring to subjects with confirmed PD diagnosis and published in English were considered. No restrictions were applied with regards to sex, age, ethnicity, sample size, follow-up duration, disease severity, and pharmacological therapy. Data were framed into a narrative review by examining the correlation between olfactory detection ability changes over time and other clinical features, focusing on disease duration, motor symptoms, cognitive impairment, non-motor symptoms, and antiparkinsonian therapy, which represent the most frequently assessed variables in both research and clinical studies on PD.

## 3. Results

**Figure 1:**
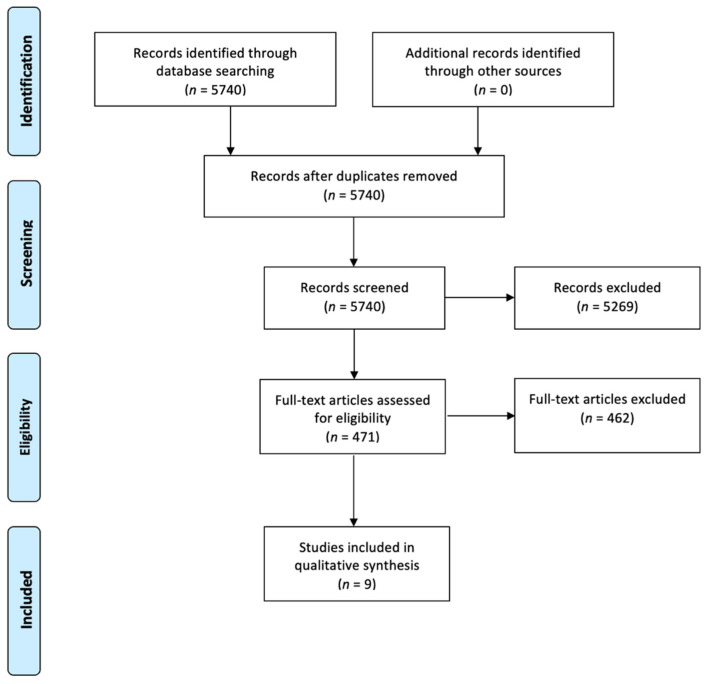
Study flow chart phases of the systematic review according to the Preferred Reporting Items for Systematic Reviews and Meta-Analyses (PRISMA) flow diagram guidelines.

Among 5740 records identified through database searching, nine longitudinal studies met the full criteria and underwent data extraction (Figure 1). At baseline assessment, six of the nine studies had no control group [24,25,26,27,28,29], while a control group was included in the others (3/9). In two of the latter studies, there was a healthy control group [30,31] and in one of them this was represented by healthy subjects and patients with Alzheimer’s disease [32]. Only two out of the nine studies investigated olfactory function in the control cohort over time [30,31] and data from the control group at baseline and follow-up were available only upon request in one study [31]. Male PD patients were the majority in eight out of the nine studies, while only in one study females were 52% of the participants [29].

Olfactory function was tested with different methods: in four studies patients, were tested with the Sniffin’ Sticks Identification Test (SST) [24,25,26,27]; in three studies, they underwent the University of Pennsylvania Smell Identification Test (UPSIT) [30,31,32]; in one study, Open Essence (OE) was performed [29]; and in one study, the Brief Smell Identification Test (B-SIT) was used [28]. Follow-up varied among studies, ranging from a minimum of five months to a maximum of eight years. The main features of the studies included in this review are summarized in Table 1.

The correlation between disease duration and smell loss was assessed in eight of the nine longitudinal studies. In three studies, the authors did not find any association between them [25,26,32]. In four studies, smell detection ability appeared to decrease in relation to disease duration [24,28,30,31]. In two of the latter studies, a significant worsening of smell function was described in the early phases of PD [24,31], while in one study, patients who were normosmic at baseline declined more than hyposmic ones at follow-up [28]. In one study, the correlation between disease duration and smell loss was not shown in the results [27].

**Figure 2:**
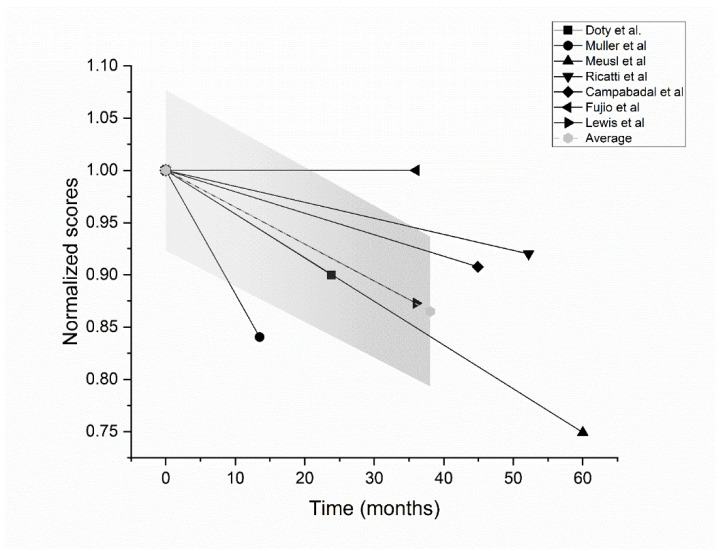
Trend of olfactory function in PD patients considering the studies providing both baseline and follow-up scores. Values from single studies are normalized by dividing them by baseline scores, so that their relative variation from a starting value of 1 is depicted. Black solid lines represent data from single studies, while their average is indicated by the grey dashed line. The grey shaded area represents the standard error of the mean.

In seven of the nine studies, both baseline and follow-up smell test scores were provided, and values from each study were normalized by dividing them by baseline scores. This analysis yielded a trend of olfactory function illustrated in Figure 2. In a mean follow-up time of 38 months, the olfactory function in PD patients declined by 14% from the first assessment.

The association between motor impairment and smell loss was assessed in six of the nine longitudinal studies. In five studies, no correlation was found between olfactory dysfunction and severity of motor symptoms assessed with the Hoehn and Yahr scale (HY) [25,26,30,31,32]. Similarly, motor impairment quantified with the UPDRS part III was not associated with smell loss in five studies [24,25,26,30,31].

The correlation between cognitive impairment and olfactory dysfunction was assessed in four of the nine studies included in the analysis. In three studies, no correlation between olfactory dysfunction and Mini-Mental State Examination (MMSE) scores was found [26,28,29]. This result was confirmed by one study where cognitive status of patients was assessed by the Montreal Cognitive Assessment (MoCA) scale [31].

The association between mood and olfactory impairment was investigated in two of the nine longitudinal studies. No association was found between smell deficits and depression, either assessed by the Beck Depression Inventory (BDI) [26] or the Hamilton Depression Rating Scale (HDRS) [30].

Only two studies analyzed the association between anti-parkinsonian therapy and smell loss. In one of them, olfactory function over time did not correlate with either medications against PD or prior thalamic surgical intervention [32]. One of the more recent studies found a relationship between Levodopa equivalent daily dose (LEDD) and smell deterioration, reporting that smell test scores correlated with a higher level of anti-parkinsonian therapy daily intake [30].

## 4. Discussion

To date, no reliable markers of disease progression in PD have been clearly investigated, and, consequently, clinical trials have often been based on subjective and/or rater-dependent measures. Therefore, our study tried to better define how smell loss evolves in PD by summarizing the results of longitudinal studies that investigated olfactory dysfunction in PD patients over time.

Although it has been known for over 40 years that smell function is compromised in PD patients [33], and despite olfactory dysfunction being a supportive criteria for PD diagnosis, according to the MDS Clinical Diagnostic Criteria for Parkinson’s Disease [34], there is still no agreement on the role of this deficit as a biomarker of disease progression and on its relationship with other clinical and demographic features of PD [35]. As PD has been also related to microglial activation, it is important to consider the future impact of the COVID-19 pandemic in terms of neurodegenerative disorders [36]. Since the olfactory bulb is a well-known target of SARS-CoV-2, researchers should focus their attention on this important relationship in order to find new evidence that is useful for other diseases as well [37,38,39,40,41].

Figure 2 shows olfactory function trend over time in PD patients considering seven of the nine studies where baseline and follow-up score values were provided. The first attempt to investigate the course of olfactory function in PD patients was made by Doty and colleagues in 1988, who observed a general decline of smell function, especially in the early stage of the disease, without a significant relationship with disease progression [32]. Those findings were confirmed by Ricatti and colleagues [27], while Muller and coworkers observed that olfactory function decreased over time among de-novo PD patients, suggesting that the correlation between disease duration and smell impairment was only detectable in the early stages of the disease [24]. Herting and coworkers observed that olfactory function was not stable over time and it did not deteriorate in a linear way [25]. A similar finding was confirmed by Meusel and colleagues and by the Domelloef’s group, who added that the decline in smell function seemed to depend on the initial olfactory status of the patient, with only patients with limited smell loss at baseline showing a clear deterioration at follow-up [26,28]. The comparison of olfactory deficits between PD patients and controls yielded contrasting results: Campabadal and others found the decrease in smell perception to be similar [30], whereas Lewis and co-workers, in a larger patient sample and with a longer study time, described a faster deterioration in PD patients [31]. The latter study [31] reported that UPSIT scores could be considered a good biomarker for PD progression, confirming results from previous cross-sectional studies [42]. Nevertheless, aging per se might be responsible for olfactory decline [43], with an approximate starting point in the fifth decade of life, and a peak between the seventh and eighth decades [44]. Consequently, the association between smell test scores and diseases duration described in some of the aforementioned studies could reflect both normal aging and progression of neurodegeneration in key brain structures. This hypothesis was supported by a magnetic resonance imaging (MRI) study that showed a significant correlation between loss of odor identification and left putamen, right thalamus, and right caudate nucleus volumes in PD patients [30].

Smell test scores correlated with a higher level of anti-parkinsonian therapy daily intake only in one study [30]. These findings were consistent with the results of a recent work that reported an association between olfactory dysfunction and higher LEDD needs [45]. Although previous studies had already described an association between cognitive decline and smell loss [46,47], none of the longitudinal studies included in this review have detected a clear relationship between olfactory impairment and MoCA/MMSE score over time. However, none of those studies investigated single domains of the aforementioned tests; therefore, the relationship between impairment in specific aspects of cognitive functions in PD patients and smell detection ability remains to be elucidated.

The aim of this review was to define a possible relationship between the progression of olfactory dysfunction and other putative clinical hallmarks of PD over time, describing the relationship between olfactory impairment and other motor and non-motor symptoms [48]. This review highlighted the fact that olfactory dysfunction in PD patients may play a key role in the evaluation of disease progression and in the assessment of patients’ quality of life. It is well known that the latter is severely impaired by olfactory dysfunction in PD patients [49] and usually decreases in relation to disease progression and worsening of other non-motor symptoms, such as cognitive abilities [50,51,52]. Our review confirms the relevance of olfactory function evaluation to provide new approaches for the early and differential diagnosis of PD. Based on these data, it would be recommended to simultaneously assess olfactory function with other motor and non-motor symptoms.

This systematic review has some limitations. The studies included were heterogenous in terms of patients’ clinical features (i.e., disease duration at baseline, clinical severity, and time between first assessment and follow-up) and methodology used to detect smell loss, so their comparison was difficult in some cases. Another limitation of this review is that PD was assessed by each study with different diagnostic criteria. Moreover, considering recent investigations [35], it is possible that distinct clinical subtypes of PD might determine a different progression of olfactory impairment. Results’ variability among studies might be related to the mentioned heterogeneity in clinical populations and to the ceiling effect observed during the late stage of disease, when olfactory detection ability seemed to reach a plateau. Moreover, the lack of longitudinal data on smell function in case-matched healthy controls or patients with other neurological disorders with possible olfactory involvement [5,8,10,11,12,53,54,55,56] in most of the studies represents a limitation to a complete understanding of the role of olfaction loss in PD.

In conclusion, olfaction seemed to decrease with a fluctuating trend over time, and not all PD patients reached a condition of anosmia. Moreover, it seemed that, particularly during the early phases of the disease, smell detection ability deteriorated more rapidly, indicating a possible association with disease progression. More studies are needed to better understand the role of olfaction as a biomarker of PD progression over time.

## Figures and Tables

**Table 1:** Features of studies included in the review.

Ref	Authors	Sample Size	Sex	Control Size	Olfactory Test	Follow-up	Clinical Features Tested
[32]	***Doty* et al.,** *1988*	PD *n* = 81 Re-tested PD *n* = 24	46 M 35 F 14 M 10 F	Matched HC form a computer-based registry and *n* = 25 patients with AD	UPSIT	Mean 23.8 ± 8.7 months (range 5–36 months)	-Age -Disease duration -H&Y -Antiparkinsonian drugs -PIT
[24]	***Muller* et al.,** *2002*	PD *n* = 5 (de-novo)	4 M 1 F	No control cohort	SST (Extended, TDI)	Mean 13.5 months (range 5–19 months)	-Age -UPDRS
[25]	***Herting* et al.,** *2008*	PD *n* = 27	22 M 5 F	No control cohort	SST (Extended, TDI)	Mean 4.4 years (range 3–6 years)	-Age -Disease duration -UPDRS III -H&Y -Clinical subtypes -Age at onset
[26]	***Meusel* et al.,** *2010*	PD *n* = 19	14 M 5 F	No control cohort	SST (Extended, TDI)	5 years	-Age -Disease duration -UPDRS -H&Y -BDI -MMSE
[27]	***Ricatti* et al.,** *2016*	PD *n* = 26	16 M 10 F	No control cohort	SST (16-stick version)	4.35 ± 0.49 years (range 3.5–5.6 years)	-H&Y -Taste (WMT, TST)
[24]	***Campabadal* et al.,** 2017	PD *n* = 25	14 M 11 F	HC *n* = 24	UPSIT	44.9 ± 5.7 months (PD) 45.9 ± 3.5 months (HC)	-Disease duration -UPDRS III -H&Y -LEDD -SPC
[28]	***Domellof* et al.,** *2017*	Baseline *n* = 125 1 year *n* = 113 3 years *n* = 92 5 years *n* = 77 8 years *n* = 27	75 M 50 F	No control cohort	B-SIT	5 years 1 year 3 years 5 years 8 years	-Age -Disease duration -UPDRS I II III IV -H&Y -PD subtypes -MMSE -MADRS
[29]	***Fujio* et al.,** *2019*	PD *n* = 56 Complete follow-up PD *n* = 42	27 M 29 F 26 M 16 F	No control cohort	OE	3 years	-MMSE
[31]	***Lewis* et al.,** *2020*	PD *n* = 125: (31 E early, 39 M middle, 55 L late stage) 1 y visit: *n* = 101 2 y visit: *n* = 90 3 y visit *n* = 80	69 M 56 F	Data available upon request to the authors	UPSIT	1 year 2 years 3 years	-Age -Education -Disease duration -UPDRS I, II, III, IV -H&Y -LEDD -MoCA -HDRS -PDQ-39

Legend: F: female; M: male; PD: Parkinson’s disease; AD: Alzheimer’s disease; HC: healthy control; UPSIT: University of Pennsylvania Smell Identification Test; SST: Sniffin’ Sticks Identification Test; B-SIT: Brief Smell Identification Test; OE: Open Essence; H&Y: Hoehn and Yahr; PIT: Picture Identification Test; UPDRS: Unified Parkinson’s Disease Rating Scale; BDI: Beck Depression Inventory; MMSE: Mini-Mental State Examination; LEDD: L-dopa equivalent daily dose; SPC: Symmetrized Percent of Change of cortical thickness on MRI; MADRS: Montgomery Asberg Depression Rating Scale; WMT: Whole Mouth Test; TST: Taste Strips Test; MoCA: Montreal Cognitive Assessment; HDRS: Hamilton Depression Rating Scale; PDQ-39: Parkinson’s Disease Questionnaire-39.

## Data Availability

Data of this study are available upon reasonable request.
